# Mediterranean diet enhances endurance training adaptation through gut microbiota-derived short-chain fatty acids

**DOI:** 10.3389/fnut.2026.1795528

**Published:** 2026-03-25

**Authors:** Cheng Lu, Chuan Liu, Gang Qin, Guangqing Song

**Affiliations:** 1 Haihua College, Liaoning Normal University, Dalian, China; 2 Department of Sports Science, Hanyang University, Seoul, Republic of Korea

**Keywords:** butyrate, endurance training, gut microbiota, Mediterranean diet, propionate, short-chain fatty acids, sports nutrition, VO_2_max

## Abstract

**Background:**

The concept of the gut microbiota and short-chain fatty acids (SCFAs) has emerged. Although the Mediterranean diet has beneficial effects on the composition of the gut microbiota and the production SCFA, it has not been shown to affect the adaptation induced by endurance exercise. This study aimed to investigate the effect of the Mediterranean diet on the production of SCFA and VO_2_max in endurance athletes.

**Methods:**

This was a 12-week randomized controlled trial among 60 competitive endurance athletes who were randomly assigned to either a Mediterranean diet group (*n* = 30) or a control group (*n* = 30). All participants followed a common training program. The composition of gut microbiota was analyzed using 16S rRNA gene sequences, and the concentration of SCFAs was analyzed using gas chromatography–mass spectrometry. The outcome measure was the change in VO_2_max. Mediation analysis was performed to examine the role of plasma SCFAs as a mediator of the association between diet and performance.

**Results:**

Fifty-five participants were included in the study. The Mediterranean diet showed a significant increase in alpha diversity (Shannon index: +11.2%, *p* = 0.002) and in the abundance of *Faecalibacterium* and *Roseburia*. Plasma propionate (+42.1%, *p* = 0.005) and butyrate (+57.9%, *p* = 0.002) increased significantly, while acetate showed a non-significant trend. VO_2_max improvement was greater in the Mediterranean diet group (2.4 ± 1.6 vs. 1.3 ± 1.4 mL/kg/min; adjusted difference: 1.1 mL/kg/min; 95% CI: 0.3–1.9; *p* = 0.006; Cohen’s d = 0.73). Plasma propionate (*r* = 0.38, *p* = 0.004) and butyrate (*r* = 0.42, *p* = 0.001) changes correlated positively with VO_2_max improvement. Mediation analysis indicated plasma propionate accounted for 23% of the dietary effect on VO_2_max. Results were consistent in intention-to-treat analysis (adjusted difference: 1.0 mL/kg/min; 95% CI: 0.2–1.8; *p* = 0.012; Cohen’s d = 0.68).

**Conclusion:**

Mediterranean diet intervention alters gut microbiota composition, increases plasma SCFA concentrations, and improves endurance exercise adaptation. These findings indicate that the gut-muscle axis may represent an intervention target for optimizing sports performance.

## 1 Introduction

Endurance exercise has been shown to stimulate a complex array of adaptations that, when taken together, increase aerobic ability; with maximal oxygen uptake (VO_2_max) being the key index of cardiorespiratory fitness and endurance ability (1). This is achieved through increased cardiac output, oxygen transport capacity, and oxidative capacity in skeletal muscle, all of which are conditioned by the stimulus for adaptation and recovery (2). Though exercise prescription and periods of training are core concepts for optimizing results of the training process, there is emerging evidence of a significant modification of these effects by nutritional interventions (3). The idea that diet is more than just a fuel source but is actually a control input that has the potential to modify signaling and metabolic programming has led to growing efforts to explore various dietary patterns that work in combination for optimizing the training response.

The gut microbiota has recently been established as a crucial regulator of metabolism, and the relationship between gut microbiota and athletic performance is now supported by a growing series of studies. Athletes can be distinguished on the basis of their unique gut microbiota, with a higher alpha diversity and abundance of taxa related to a positive metabolism when compared with non-active persons (4). Of the metabolites generated in the gut, short-chain fatty acids (SCFAs)—primarily acetate, propionate, and butyrate—have gained special prominence based on their potential for influencing metabolic function (5). A groundbreaking study showed that *Veillonella atypica*, which was enriched in marathon runners following exercise, could convert lactate into propionate, with beneficial effects on treadmill performance in mice, thus establishing a direct mechanism between microbial metabolism and exercise performance. However, recent studies have identified that SCFAs regulate muscle metabolism through various mechanisms, such as activating AMP-activated protein kinase (AMPK), stimulating mitochondrial biogenesis, and regulating glucose uptake (6, 7). Thus, the microbial and metabolic products of the gut have emerged as promising targets for interventions aiming to enhance adaptations to endurance exercise (8).

The Mediterranean diet, which is characterized by a high intake of plant foods, olive oil, and fiber, has been strongly associated with desirable changes in the composition of the gut microbiota (9, 10). Efficacy studies show that the adherent population of this dietary profile increases the concentration of SCFA-producing bacteria such as *Faecalibacterium prausnitzii* and *Roseburia* species, while elevating fecal and circulating SCFA concentrations (11, 12). These changes in microbiota and metabolism are associated with a reduction in the severity of systemic inflammation and improvement in the values of metabolic health (13). Given the established role of SCFAs in modulating muscle function and the Mediterranean diet’s ability to increase concentrations of SCFAs, one can postulate that complying with the Mediterranean diet could improve endurance training responses through microbial metabolites. Nevertheless, this hypothesis has neither been validated nor rejected among sport participants. Existing studies in progress regarding interactions between diet and microbiota are carried out among sedentary individuals or clinical patients because there are relatively few data among sport participants regarding athlete microbiota (14). The limited number of studies on dietary interventions in athletes has almost entirely focused on ketogenic or protein-rich diets, with little thought given to the fiber-rich diet and the potential for the SCFA production (15). It seems that the endurance performance area is a missed opportunity for the application of microbiome science to performance enhancement.

This study addresses the knowledge gap by conducting a 12-week randomized clinical trial to investigate the impact of the Mediterranean diet on the intestinal microbiota profile and SCFA pattern, as well as the endurance training adaptations in competitive endurance athletes. Three interrelated hypotheses were addressed in the study: the Mediterranean diet increases the abundance of SCFA-producing bacteria, particularly *Faecalibacterium* and *Roseburia*, in the gut microbiota of endurance athletes (H1); these microbiota changes result in elevated SCFA concentrations in both fecal and plasma samples (H2); and circulating SCFA levels are positively associated with improvements in VO_2_max and serve as mediators of the dietary effect on training adaptation (H3). To examine these hypotheses, the participants were allocated to either the standardized Mediterranean diet or the control diet but with the same periodic training program. Analysis of the gut microbiota was performed by 16S rRNA gene sequencing, while the concentration of SCFA was determined by targeted gas chromatography–mass spectrometry analysis of both fecal and plasma specimens. The main outcome measure was changed in VO_2_max, with mediation analysis performed to assess the impact of SCFAs on the relationship between diet and aerobic adaptation. This study offers mechanistic insights into the relationship between the gut and muscle in athletic subjects that could lead to a focus on nutritional interventions based on the microbiome in endurance events. Circulating plasma SCFAs were prioritized as the primary metabolite outcome over fecal SCFAs because plasma concentrations reflect the fraction absorbed into the portal circulation and systemically available to peripheral tissues including skeletal muscle, and are therefore more directly relevant to the proposed gut-muscle axis mechanism than fecal concentrations, which reflect luminal production prior to absorption. Although fecal SCFAs were also measured as a secondary outcome, the ongoing debate in the field regarding the relative informativeness of fecal versus plasma SCFAs as markers of systemic metabolic adaptation is acknowledged as a contextual limitation of this approach. It should also be noted that substantial inter-individual variability exists in gut microbiota composition and metabolic responsiveness to dietary interventions, arising from differences in baseline microbiota architecture, host genetics, and prior dietary history; such variability may attenuate the average treatment effect and may help explain why a subset of participants in the present study exhibited limited plasma SCFA responses despite confirmed dietary adherence.

## 2 Methods

### 2.1 Study design and participants

This study used a parallel group, randomized controlled clinical trial design lasting a total of 12 weeks to assess the impact of a Mediterranean diet treatment on composition, short-chain fatty acid profiles, and endurance training. The study protocol was approved by the Institutional Review Board of Hanyang University (Approval No. IRB-0386-2025). All procedures adhered to the principles outlined in the Declaration of Helsinki, and written informed consent was obtained from each participant prior to enrollment.

The competitive endurance participants were sought from local running clubs, cycling groups, and triathlon clubs through advertisements. The inclusion criteria included male and female participants aged 18- to 35-years-old who engaged in structured endurance training for at least 2 years with a weekly training duration of 8 h. The inclusion criterion for maximal aerobic exercise was established at VO_2_max exceeding 50 mL/kg/min in males, while in females, it was VO_2_max exceeding 45 mL/kg/min. Excluded from the study were athletes taking antibiotics or probiotic within the last 3 months, showing symptoms of gastrointestinal tract disease, such as inflammatory bowel disease or irritable bowel syndrome, strictly following a ketogenic diet, vegetarian diet, or being injured to the point of suspending their training program within the last 6 months.

Sample size calculation was performed with the G*Power software (version 3.1), with the purpose of determining between-group differences on the VO2max value. Assuming a medium-to-large effect size (Cohen’s d = 0.8) derived from previous nutritional intervention studies in athletes, a two-tailed alpha of 0.05, and statistical power of 80%, a minimum of 26 participants per group was required (16). In order to adjust to the possible dropout rate of about 15%, the target sample size of each group was set at 30, resulting in a total of 60 athletes.

Participants were stratified by sex and VO_2_max at baseline, and then randomly assigned to the Mediterranean dietary pattern group or the control group. This allocation was made using a computer-generated random permuted block allocation with variable blocks of sizes four and six. The allocation sequence was created by an independent statistician, and concealment of allocation was ensured using a closed envelope system in which the allocation was opened only after completion of the baseline measurements. Blinding of participants and study personnel was not possible due to the nature of the dietary interventions; however, the outcome evaluators assessing the exercise tests and the laboratory analysis were blinded throughout the study. Evaluations were carried out at baseline (T0), the midpoint of the intervention (T6, week 6), and upon completion of the 12-week intervention period (T12).

### 2.2 Dietary intervention and training protocol

Participants in the Mediterranean diet group were provided with personalized diets created by registered dietitians in adherence to the traditional Mediterranean diet pattern (17). This included focusing on whole grains, beans, vegetables, fruits, nuts, and using extra virgin olive oil as the primary source of fats, moderation of fish and poultry products, while restricting red meats, processed meats, and refined sugars. The daily fiber intake was fixed at 30–35 grams to ensure the growth of SCFA-producing gut bacteria. Adherence to the dietary regime was measured by the 14-item Mediterranean Diet Adherence Screener (MEDAS), where a score of 9 and above was taken as good adherence to the regime (11). Participants in this study group received personalized counseling from diet initially as well as at periodic intervals of 2 weeks, wherein their meal plans were discussed, recipes were given, and plans to adhere to the regime in the long term were devised. A starter kit of extra virgin olive oil, whole grains, as well as dried legumes was given to aid in the execution of the already advised dietary regime. Participants were instructed to prepare their own meals at home following the personalized meal plans provided by the dietitians; no pre-prepared or catered meals were supplied by the research team. Each participant received a written food guide detailing recommended foods, portion sizes, and meal composition examples, supplemented with photographic references to support independent meal preparation at home.

The control group participants were asked to maintain their usual eating habits during the intervention phase. To provide equal attention to the groups, control participants were given general information on nutritional aspects, which includes hydration and proper macronutrient intake in relation to their training schedule, without giving much emphasis to fiber and Mediterranean diet (3). The control group also underwent nutrition consultations on baseline and at week 6 and 12 to determine their stability in food habits and to respond to general nutritional concerns unrelated to the intervention.

The dietary intake was assessed through 3-day food records performed at baseline, at week 6, and at week 12. Foods and drinks consumed during two weekdays and one weekend day were recorded. These were analyzed using the Computer Aided Nutritional analysis program (CAN-Pro, version 5.0; Korean Nutrition Society, Seoul, Republic of Korea) to assess total energy, macronutrient ratio, and dietary fiber. The MEDAS questionnaire was also employed at the same points to evaluate changes in levels of Mediterranean diet adherence. For each assessment point, dietary variables were calculated as the mean of the three recorded days to account for day-to-day variability in intake. These mean values were then used as the dietary exposure variable in all subsequent analyses. Day-to-day variability in carbohydrate and fiber intake within each recording period was examined descriptively and found to be within acceptable ranges (coefficient of variation <20% for fiber intake), supporting the representativeness of the three-day mean as an index of habitual dietary intake at each time point.

All participants completed a standardized 12-week periodized endurance training program designed by professional coaches specialized in endurance sports. The program included three mesocycles, each lasting 4 weeks, which included progressive changes in the volume and intensity of the training. The weekly endurance training included four to six sessions, which included long distance runs or rides, tempo sessions performed at the intensity corresponding to the blood lactate threshold, and interval workouts performed at 90–100% maximal heart rate. Training volumes were then individualized for each participant based on fitness level and competitive schedule, with goals of exercise duration between 8 and 12 h per week. To ensure that the group conditions remained consistent across the two study groups, the exercise diaries were completed each week using a mobile application, with feedback obtained from the coaching staff whenever there were deviations of over 10 percent from the programmed exercise protocol. Heart rate monitoring was performed throughout each session to ensure that the intensity of exercise was confirmed.

Energy needs were individualized to match training requirements and maintain individual body weight stability throughout the experiment, with both groups given the same training on how to manage energy needs. However, protein was set at 1.4–1.6 grams per kilogram of body weight per day to adequately meet training needs while excluding the impact of the Mediterranean diet pattern on gut microbiota and SCFA production.

### 2.3 Sample collection and processing

Blood samples were collected at three distinct points in time in this study: baseline (T0), week 6 (T6), and week 12 (T12). The participants were asked to avoid intense exercise in the 48 h leading up to blood sampling as well as alcohol in the 24 h prior to blood collection. Blood sampling took place in the morning after at least a 10-h fast to avoid the possibility of variations in metabolite levels due to natural rhythms.

Fecal specimens were self-collected by the participants at home on the morning of their allocated laboratory session, using sterilized collection kits procured by the study team. The kit contained a specimen container with a spoon-lid design, an oxygen-absorbing package to protect against oxygenation, and articulated step-by-step written guidelines on how to undertake the process. The participants were asked to ensure there was no urine contamination and to collect the specimens from different areas of the stool. The samples were transported to the research lab within 2 h of sampling, using insulated containers and ice packs to keep the temperature below 4 °C. The fecal samples were aliquoted into cryovials under aseptic conditions upon arrival. They were then placed in −80 °C units for later examination of DNA and metabolic profiling. Ready-made questionnaires were used to assess bowel habits, stool scores based on the Bristol stool scale, as well as the presence of any symptoms pertaining to the gut. Bristol Stool Scale (BSS) scores were recorded at T0, T6, and T12. At baseline, median BSS scores were comparable between groups [Mediterranean diet: 3.0 (IQR: 3.0–4.0); control: 3.0 (IQR: 3.0–4.0); *p* = 0.84]. In the Mediterranean diet group, median BSS scores were 3.0 (IQR: 3.0–4.0) at T6 and 4.0 (IQR: 3.0–4.0) at T12, reflecting a modest shift toward type 4 (normal formed stool) consistent with increased dietary fiber intake. In the control group, scores remained stable across all time points [T6: 3.0 (IQR: 3.0–4.0); T12: 3.0 (IQR: 3.0–4.0)]. The group-by-time interaction did not reach statistical significance (*p* = 0.18). No participant reported clinically meaningful gastrointestinal symptoms or adverse events attributable to the dietary intervention throughout the study period.

Venous blood samples were taken in the laboratory by trained phlebotomists using standard venipuncture techniques. About 20 mL of blood samples were taken using EDTA-coated tubes for separating plasma. Serum separation samples were taken using serum separator tubes. These samples were processed within 30 min of being taken by centrifugation at 3000 × *g* for 10 min at 4 °C. Plasma and serum aliquots were then placed into labeled cryovials and stored at −80 °C until bulk analysis could be completed. Aliquots of the samples were set aside for analysis of short-chain fatty acids, inflammation, and further exploratory analysis.

### 2.4 Gut microbiota and metabolome analysis

The total bacterial DNA was obtained from fecal samples using the QIAamp PowerFecal Pro DNA Kit following the manufacturer’s protocol with slight modifications. Bead beating was performed for 10 min, where Gram-positive bacteria have enhanced lysis. The quality and purity of obtained DNA were checked using a NanoDrop spectrophotometer (Thermo Fisher Scientific, USA), where DNA purity was considered acceptable if it met A260/A280 values ranging from 1.8 to 2.0. The V3-V4 hypervariable regions of the 16S rRNA gene were amplified using universal primers 341F (5’-CCTACGGGNGGCWGCAG-3′) and 805R (5’-GACTACHVGGGTATCTAATCC-3′) with Illumina adapter overhangs. Polymerase chain reaction conditions included an initial denaturation of 3 min at 95 °C, followed by 25 cycles of denaturation for 30 s at 95 °C, annealing for 30 s at 55 °C, and extension for 30 s at 72 °C, and a final extension of 5 min at 72 °C. The amplicon libraries were purified, normalized, and mixed in equal molar concentrations before being subjected to Illumina MiSeq (Illumina, USA) using a 2 × 300 bp paired-end configuration.

The raw sequencing files were analyzed using the QIIME2 (version 2023.5) following established pipelines (18). The removal of the primer sequences was performed by the Cutadapt tool, along with the workflow of DADA2 for the filtering, removal of chimerical sequences, and the production of the amplicon sequence variants (19). The assignments were performed using the SILVA 138.1 marker gene reference database by the naive Bayes classifier, and the sequence variants which had fewer than 10 total reads were eliminated. For alpha-diversity analysis, the Shannon index and Chao1 richness index were calculated on rarefied samples with 20,000 reads per sample. For beta-diversity analysis, Bray-Curtis dissimilarities were calculated, and the resulting data were plotted using Principal Coordinates Analysis (PCoA). Linear Discriminant Analysis of Effect Size (LEfSe) was used for differential abundance analysis between groups, with an LDA score threshold of 2.0 (20).

Analysis of fecal and plasma samples of the SCFAs present in these samples is carried out by using gas chromatography–mass spectrometry techniques (GC–MS). To analyze the SCFA in fecal samples, 100 mg of thawed feces was mixed in 1 mL of acidified water using 0.5% phosphoric acid with an internal standard (4-methylvaleric acid). After centrifuging the mixture at 15,000 x g at 4 °C for 15 min, the supernatant was separated using ethyl acetate in a liquid–liquid separation technique. Plasma samples underwent protein precipitation with acetonitrile followed by derivatization with propyl chloroformate to enhance volatility and detection sensitivity. Prepared extracts were analyzed on a GC–MS system (Agilent 7890B-5977B, Agilent Technologies, USA) equipped with a DB-FFAP capillary column (30 m × 0.25 mm × 0.25 μm). Target analytes included acetate, propionate, butyrate, isobutyrate, valerate, and isovalerate. Quantification of results utilized calibration curves created from standards. Quality control samples were inserted variably throughout a set of analyses, with acceptability criteria being coefficients of variation under 15%. All gut microbiota 16S rRNA amplicon sequencing and fecal SCFA metabolomic analyses were conducted by Macrogen Inc. (Seoul, Republic of Korea), a CAP-accredited and ISO 9001:2015-certified genomics and sequencing service provider. Plasma SCFA quantification by GC–MS, along with blood biochemistry and inflammatory marker analyses, were performed at the Clinical Laboratory of Hanyang University Medical Center (Seoul, Republic of Korea), a ISO 15189-accredited medical laboratory affiliated with the study institution.

### 2.5 Exercise testing and statistical analysis

Maximal oxygen uptake measurements took place using a graded exercise test to voluntary exhaustion conducted either on a motorized treadmill (h/p/cosmos, Germany) or an electromagnetically braked cycle ergometer (Lode Excalibur Sport, Netherlands), depending on the dominant sport discipline in which each individual took part. The treadmill test began at 8 km/h with a 1 km/h increase per minute, whereas in the cycle test, it started at 100 W with an increase every 25 W per minute. Expired gases were continuously analyzed using a metabolic cart (Cosmed Quark CPET, Italy) after calibration with reference gases just before testing. Heart rates were measured using telemetry (Polar H10, Finland), while ratings of perceived exertion were recorded after the completion of each stage of exercise. To minimize within-subject variability across repeated test sessions, pre-test conditions were standardized: all exercise tests were conducted at the same time of day (08:00–10:00) for each participant, participants were instructed to refrain from vigorous training in the 48 h preceding each test session, to abstain from alcohol for 24 h prior, and to arrive in a 10-h fasted state, consistent with the blood sampling protocol applied at each assessment point. VO_2_max was expressed as the highest 30-s moving average value for oxygen consumption reached during the test, and achievement thereof was confirmed when at least two of the following were satisfied: respiratory exchange ratio exceeding 1.10, heart rate within 10 beats per minute of the age-predicted maximum, or attainment of a plateau for oxygen uptake despite an increase in workload. Time to exhaustion was used as a secondary performance measure. Lactate threshold was analyzed using capillary blood samples taken from the fingertip during the final 15 s of each exercise level using a portable lactate analyzer (Lactate Pro 2, Arkray, Japan), with the intensity of exercise at which blood lactate concentration reaches a fixed level of 4 mmol/L.

Statistical analyses included R (version 4.3.1) and SPSS (version 28.0). Baseline differences between groups in both continuous and categorical variables were determined using independent samples t-tests and chi-square tests, respectively. The difference in change in VO_2_max from baseline to week 12 between groups was determined using ANCOVA in the primary analysis with covariates VO_2_max at baseline, age, sex, and body mass index. These covariates were selected *a priori* based on their established associations with VO₂max and SCFA metabolism: baseline VO₂max was included to control for regression to the mean, while age, sex, and BMI were included as physiological determinants of both aerobic capacity and gut microbiota composition that were not perfectly balanced by stratified randomization alone. The secondary continuous outcomes were evaluated using equivalent ANCOVA models. To supplement the primary between-group ANCOVA comparisons, repeated measures ANOVA was applied to characterize within-group temporal changes at each time point (T0, T6, T12), and linear mixed-effects models were used to assess group-by-time interaction effects across the full intervention period; both sets of statistics are reported alongside ANCOVA results in Tables 1, 2. To determine the differences in microbiota composition, the permutation multivariate analysis of variance (PERMANOVA) was conducted using the Bray–Curtis dissimilarity matrices. The permutation test had 999 permutations and was conducted using the vegan package in R. Spearman’s rank correlation coefficients were used to examine the relationships between concentrations of the SCFA and the outcomes of performance. Mediation analysis was also employed to examine the degree to which changes in plasma concentrations of SCFA contributed to the relationship between the dietary intervention and improvements in VO_2_max, utilizing the mediation package with 5,000 bootstrap resamples to estimate indirect effects and 95% confidence intervals. In order to adjust the false discovery rate, the Benjamini-Hochberg correction method was applied to the results of the microbiome analysis. For all the analysis of variances, the level of significance applied was *p* < 0.05, and effect sizes (Cohen’s d for between-group comparisons) were used together with the *p*-values. The calculation of the effect size was performed using the formula proposed by Cohen, employing the pooled standard deviations. The thresholds of 0.2, 0.5, and 0.8 were considered small, medium, and large, respectively. The confidence interval for the effect size was calculated using the non-central t-distribution. The analysis was carried out both by intention-to-treat analysis and per-protocol. Missing outcome data arising from participant withdrawal were handled using the last observation carried forward (LOCF) method within the intention-to-treat framework: the most recently observed value for each outcome variable was carried forward to impute missing assessments. Five participants withdrew during the intervention (three from the Mediterranean diet group, two from the control group; see Results), all of whom had at least T0 data available. Sensitivity analyses confirmed that findings were not materially altered by inclusion of imputed values. Although both male and female participants were included, the study was not powered to support sex-stratified analyses: with a total sample of 60 participants, post-hoc subdivision by sex would yield subgroups of approximately 15 per cell, providing insufficient statistical power to detect clinically meaningful sex-by-intervention interactions. Exploratory sex-by-intervention interaction terms were therefore not examined, and findings should be interpreted as applying to a mixed-sex endurance athlete population.

**Table 1:** Dietary intake and Mediterranean diet adherence during the intervention.

Variable	Group	Baseline (T0)	Week 6 (T6)	Week 12 (T12)	p (time)	p (group × time)
MEDAS score (0–14)	Mediterranean	5.8 ± 1.9	9.6 ± 1.8	10.4 ± 1.7	<0.001	<0.001
	Control	5.6 ± 2.1	5.7 ± 2.0	5.9 ± 2.0	0.42	
Dietary fiber (g/day)	Mediterranean	18.2 ± 4.6	29.4 ± 5.8	32.8 ± 5.1	<0.001	<0.001
	Control	17.8 ± 5.2	18.1 ± 4.7	18.5 ± 4.9	0.31	
Energy intake (kcal/day)	Mediterranean	2,856 ± 412	2,912 ± 398	2,945 ± 425	0.18	0.72
	Control	2,798 ± 445	2,834 ± 418	2,867 ± 452	0.24	
Carbohydrate (% energy)	Mediterranean	48.2 ± 5.4	50.8 ± 4.6	51.4 ± 4.2	0.02	0.03
	Control	47.5 ± 6.1	47.8 ± 5.8	48.1 ± 5.5	0.58	
Protein (% energy)	Mediterranean	18.4 ± 2.8	17.2 ± 2.4	16.8 ± 2.2	0.04	0.08
	Control	18.9 ± 3.1	18.6 ± 2.9	18.4 ± 3.0	0.42	
Fat (% energy)	Mediterranean	33.4 ± 4.2	32.0 ± 3.8	31.8 ± 3.5	0.12	0.24
	Control	33.6 ± 4.8	33.6 ± 4.5	33.5 ± 4.6	0.95	
Protein intake (g/kg/day)	Mediterranean	1.52 ± 0.24	1.48 ± 0.22	1.46 ± 0.20	0.28	0.65
	Control	1.54 ± 0.28	1.52 ± 0.26	1.51 ± 0.25	0.54	
Weekly training volume (h)	Mediterranean	10.2 ± 1.8	10.5 ± 2.0	10.8 ± 1.9	0.32	0.86
	Control	9.9 ± 2.1	10.3 ± 2.2	10.6 ± 2.0	0.28	
Mean HR (% HRmax)	Mediterranean	73.2 ± 5.8	74.1 ± 5.5	74.8 ± 5.2	0.38	0.78
	Control	72.5 ± 6.1	73.8 ± 5.9	74.2 ± 5.6	0.42	
Weekly TRIMP (AU)	Mediterranean	425 ± 85	458 ± 78	482 ± 82	0.02	0.88
	Control	418 ± 92	452 ± 88	476 ± 90	0.03	
Session RPE (1–10)	Mediterranean	5.6 ± 1.3	5.8 ± 1.2	5.9 ± 1.1	0.35	0.72
	Control	5.5 ± 1.4	5.6 ± 1.2	5.7 ± 1.3	0.42	
Training compliance (%)	Mediterranean	—	95.2 ± 5.4	94.2 ± 5.8	—	0.81
	Control	—	94.5 ± 5.8	93.8 ± 6.2	—	

Data are presented as mean ± SD. *p* (time) from repeated measures ANOVA within each group; *p* (group × time) from mixed-effects model for group-by-time interaction. MEDAS, Mediterranean Diet Adherence Screener; HR, heart rate; HRmax, maximal heart rate; TRIMP, training impulse; AU, arbitrary units; RPE, rating of perceived exertion.

**Table 2:** Fecal and plasma short-chain fatty acid concentrations.

Variable	Group	Baseline (T0)	Week 6 (T6)	Week 12 (T12)	Change (%)	p (time)	*p* (group × time)
Fecal SCFA (μmol/g wet weight)							
Total SCFA	Mediterranean	68.4 ± 12.8	74.8 ± 14.1	82.6 ± 15.2	+20.8	0.004	0.008
	Control	67.8 ± 13.2	68.4 ± 12.9	69.1 ± 13.5	+1.9	0.71	
Acetate	Mediterranean	42.1 ± 8.2	44.8 ± 8.9	48.2 ± 9.8	+14.5	0.062	0.09
	Control	41.8 ± 8.5	42.0 ± 8.3	42.2 ± 8.6	+1.0	0.82	
Propionate	Mediterranean	14.8 ± 3.4	17.1 ± 3.9	19.6 ± 4.2	+32.4	0.002	0.003
	Control	14.5 ± 3.2	14.6 ± 3.4	14.8 ± 3.5	+2.1	0.68	
Butyrate	Mediterranean	11.5 ± 2.8	12.9 ± 3.1	14.8 ± 3.4	+28.7	0.008	0.012
	Control	11.5 ± 2.9	11.8 ± 2.8	11.9 ± 3.0	+3.5	0.58	
Isobutyrate	Mediterranean	1.8 ± 0.5	1.8 ± 0.5	1.9 ± 0.6	+5.6	0.42	0.68
	Control	1.9 ± 0.5	1.9 ± 0.5	1.9 ± 0.5	0.0	0.95	
Valerate	Mediterranean	1.4 ± 0.4	1.4 ± 0.4	1.5 ± 0.5	+7.1	0.38	0.52
	Control	1.4 ± 0.4	1.4 ± 0.4	1.4 ± 0.4	0.0	0.91	
Isovalerate	Mediterranean	1.2 ± 0.3	1.2 ± 0.3	1.2 ± 0.4	0.0	0.85	0.78
	Control	1.2 ± 0.3	1.2 ± 0.3	1.2 ± 0.3	0.0	0.96	
Plasma SCFA (μmol/L)							
Acetate	Mediterranean	45.2 ± 8.5	47.4 ± 9.2	50.8 ± 10.2	+12.4	0.08	0.12
	Control	44.8 ± 8.8	45.0 ± 8.7	45.6 ± 9.2	+1.8	0.68	
Propionate	Mediterranean	3.8 ± 0.9	4.5 ± 1.1	5.4 ± 1.2	+42.1	0.001	0.005
	Control	3.7 ± 0.8	3.8 ± 0.9	3.9 ± 0.9	+5.4	0.48	
Butyrate	Mediterranean	1.9 ± 0.5	2.3 ± 0.6	3.0 ± 0.8	+57.9	<0.001	0.002
	Control	1.8 ± 0.4	1.9 ± 0.5	2.0 ± 0.5	+11.1	0.35	

Data are presented as mean ± SD. Change (%) represents the percentage change from baseline to week 12. *p* (time) from repeated measures ANOVA within each group; p (group × time) from mixed-effects model for group-by-time interaction. SCFA, short-chain fatty acids.

## 3 Results

### 3.1 Participant characteristics and dietary compliance

**Figure 1:**
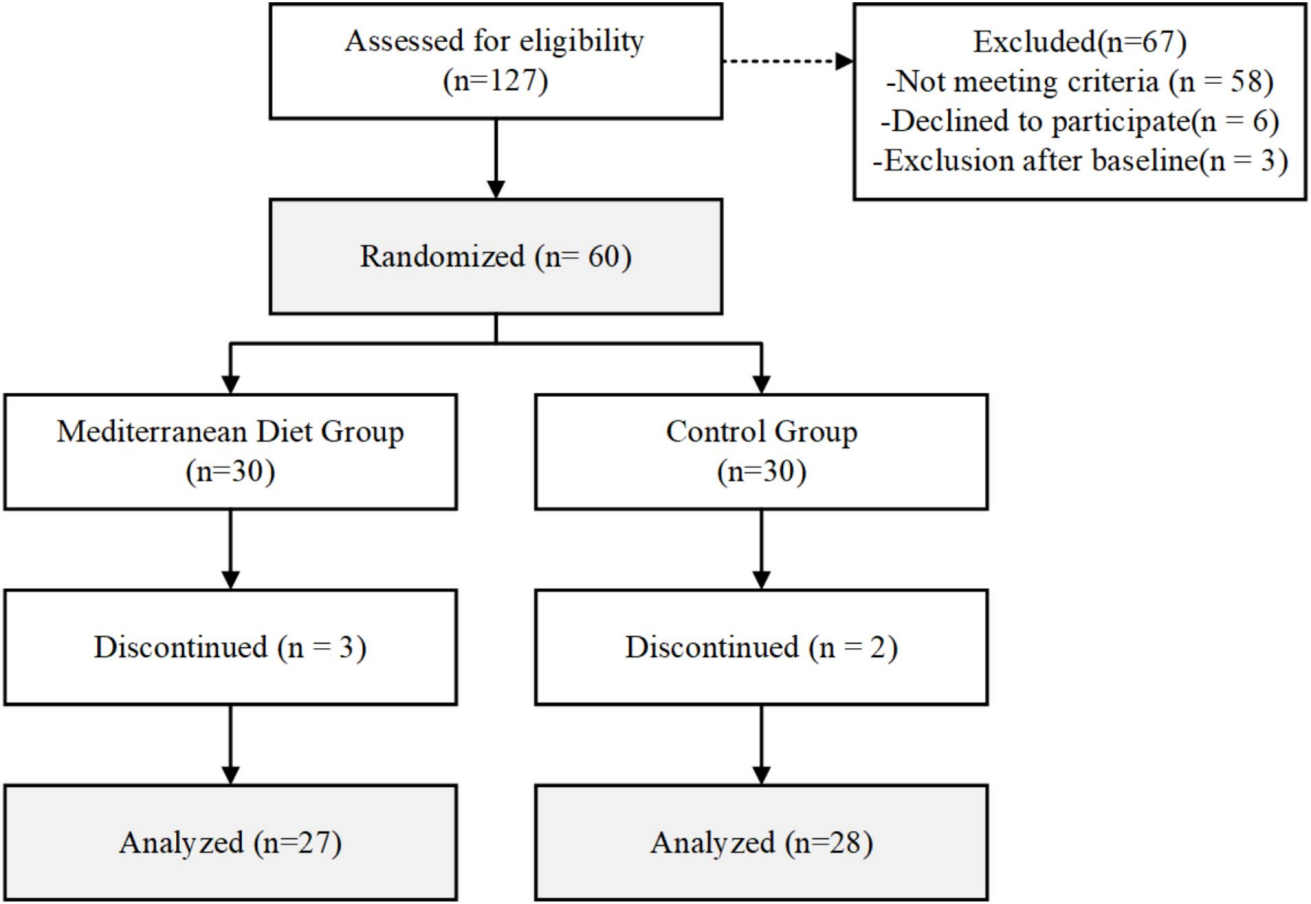
CONSORT flow diagram.

Between March and June 2024, a total of 127 endurance athletes were screened for eligibility through online questionnaires and telephone interviews. Of these, 58 individuals did not meet the inclusion criteria, primarily due to insufficient training volume (*n* = 23), VO_2_max values below the required thresholds (*n* = 18), or recent antibiotic use (*n* = 11). An additional six athletes declined to participate after learning about the dietary requirements of the study. The remaining 63 eligible participants provided written informed consent and underwent baseline assessments. Three participants were excluded prior to randomization (one for VO₂max below threshold on formal testing, one for abnormal blood parameters, and one who withdrew for personal reasons), leaving 60 athletes who were enrolled and randomly allocated to either the Mediterranean diet group (*n* = 30) or the control group (*n* = 30). The participant flow throughout the study is illustrated in Figure 1.

During the 12-week intervention period, five participants withdrew from the study: three from the Mediterranean diet group (one due to injury unrelated to the intervention, one due to scheduling conflicts, and one lost to follow-up) and two from the control group (both due to personal reasons). Consequently, 27 participants in the Mediterranean diet group and 28 participants in the control group completed the full intervention and were included in the per-protocol analysis. Intention-to-treat analysis using last observation carried forward for the five participants who discontinued (*n* = 60) yielded results consistent with the per-protocol analysis. The between-group difference in VO_2_max change remained statistically significant in the ITT analysis (adjusted mean difference: 1.0 mL/kg/min; 95% CI: 0.2–1.8; *p* = 0.012), confirming the robustness of the primary findings. All subsequent results are reported for the per-protocol population unless otherwise specified.

Baseline characteristics of participants are presented in Table 3. The two groups were well-matched at baseline with no statistically significant differences in age, sex distribution, body mass index, training experience, weekly training volume, or VO_2_max values. Both groups exhibited comparable dietary patterns at baseline, with similar energy intake, macronutrient distribution, and Mediterranean diet adherence scores.

**Table 3:** Baseline characteristics of participants.

Characteristic	Mediterranean diet group (*n* = 30)	Control group (*n* = 30)	*p*-value
Demographics			
Age (years)	26.4 ± 4.8	25.9 ± 5.1	0.68
Sex, male/female	18/12	17/13	0.79
Body mass (kg)	68.2 ± 8.4	67.5 ± 9.1	0.75
Height (cm)	174.3 ± 7.8	173.6 ± 8.2	0.72
Body mass index (kg/m^2^)	22.4 ± 1.8	22.3 ± 2.0	0.84
Training Background			
Training experience (years)	5.8 ± 2.4	6.1 ± 2.7	0.64
Weekly training volume (h/week)	10.2 ± 1.8	9.9 ± 2.1	0.55
Primary sport (running/cycling/triathlon)	14/10/6	13/11/6	0.92
Physiological parameters			
VO_2_max (ml/kg/min)	54.8 ± 4.2	55.3 ± 4.6	0.66
Resting heart rate (bpm)	52.4 ± 5.8	51.8 ± 6.2	0.69
Lactate threshold (% VO_2_max)	78.2 ± 4.5	77.8 ± 4.9	0.74
Baseline dietary intake			
Energy intake (kcal/day)	2,856 ± 412	2,798 ± 445	0.60
Carbohydrate (% energy)	48.2 ± 5.4	47.5 ± 6.1	0.63
Protein (% energy)	18.4 ± 2.8	18.9 ± 3.1	0.51
Fat (% energy)	33.4 ± 4.2	33.6 ± 4.8	0.86
Dietary fiber (g/day)	18.2 ± 4.6	17.8 ± 5.2	0.74
MEDAS score (0–14)	5.8 ± 1.9	5.6 ± 2.1	0.70

Data are presented as mean ± SD or *n*. *p*-values from independent samples *t*-tests for continuous variables and chi-square tests for categorical variables. MEDAS, Mediterranean Diet Adherence Screener; VO_2_max, maximal oxygen uptake.

Dietary compliance data are summarized in Table 1. Participants in the Mediterranean diet group demonstrated substantial improvements in adherence to the prescribed dietary pattern, with MEDAS scores increasing from 5.8 ± 1.9 at baseline to 10.4 ± 1.7 at week 12 (*p* < 0.001), representing an 79% improvement. In contrast, the control group maintained stable MEDAS scores throughout the intervention (baseline: 5.6 ± 2.1; week 12: 5.9 ± 2.0; *p* = 0.42). Daily dietary fiber intake in the Mediterranean diet group increased significantly from 18.2 ± 4.6 g/day to 32.8 ± 5.1 g/day (*p* < 0.001), reaching the target range of 30–35 g/day, while the control group showed no significant change (baseline: 17.8 ± 5.2 g/day; week 12: 18.5 ± 4.9 g/day; *p* = 0.31).

Training load monitoring confirmed comparable exercise stimulus between groups throughout the intervention (Table 1). Mean training heart rate expressed as percentage of maximal heart rate was similar in the Mediterranean diet (74.8 ± 5.2%) and control (74.2 ± 5.6%) groups at week 12 (*p* = 0.78 for group × time interaction). Weekly training impulse (TRIMP) increased progressively in both groups consistent with the periodized design, with no between-group differences (*p* = 0.88). Session ratings of perceived exertion averaged 5.8 ± 1.2 and 5.6 ± 1.3 in the Mediterranean diet and control groups, respectively (*p* = 0.72). Training compliance exceeded 90% in both groups throughout the intervention, confirming that any observed effects on performance outcomes could be attributed to the dietary intervention rather than differences in training load.

### 3.2 Gut microbiota responses to Mediterranean diet

After quality filtering and chimera removal, 16S rRNA gene sequencing yielded an average of 42,568 ± 8,124 high-quality reads per sample. A total of 2,847 amplicon sequence variants (ASVs) were identified across all samples, with adequate sequencing depth confirmed by rarefaction curve analysis.

Alpha diversity metrics revealed divergent trajectories between groups over the intervention period. The Shannon diversity index in the Mediterranean diet group increased significantly from baseline (4.12 ± 0.45) to week 12 (4.58 ± 0.52), representing an 11.2% improvement (*p* = 0.002). The control group exhibited no significant change in Shannon index throughout the study (baseline: 4.08 ± 0.51; week 12: 4.15 ± 0.48; *p* = 0.58). The between-group difference in Shannon index change was substantial (Cohen’s d = 1.18). A similar pattern was observed for species richness estimated by the Chao1 index, with the Mediterranean diet group showing a 15.8% increase (*p* = 0.008) compared to a non-significant 3.2% change in controls (*p* = 0.42). The group-by-time interaction was statistically significant for both Shannon (*p* = 0.006; d = 1.18) and Chao1 (*p* = 0.012; d = 0.95) indices.

**Figure 2:**
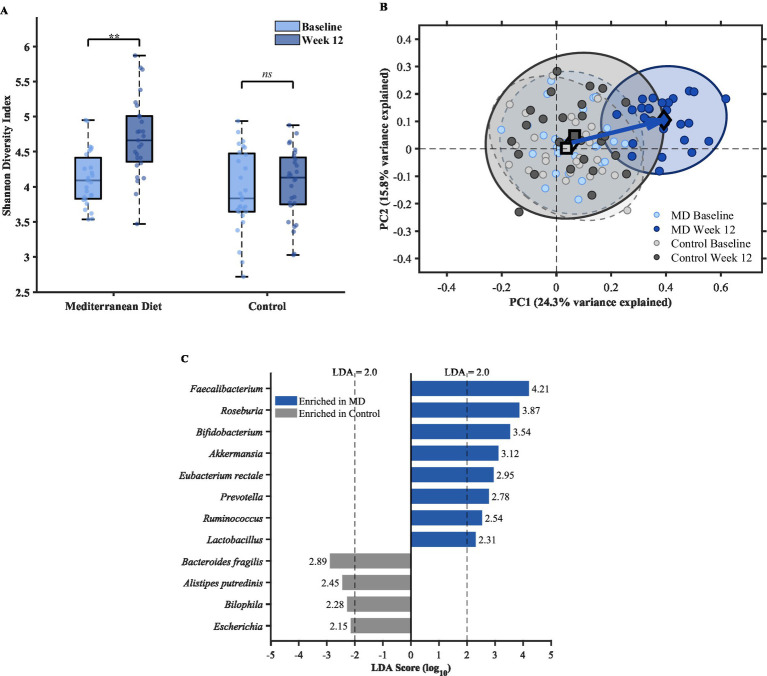
Gut microbiota responses to Mediterranean diet intervention **(A)** Shannon diversity index at baseline and week 12 in Mediterranean diet (MD) and control groups. Box plots display median, interquartile range, and individual data points. *p* < 0.01 for within-group comparison; NS, not significant. **(B)** Principal coordinates analysis (PCoA) based on Bray–Curtis dissimilarity illustrating shifts in gut microbiota composition. Each point represents an individual sample; ellipses indicate 95% confidence intervals. Diamonds and squares denote group centroids for MD and control groups, respectively. Arrow indicates the direction of microbiota shift in the MD group. PERMANOVA: *R*^2^ = 0.089, *p* = 0.001. **(C)** Linear discriminant analysis effect size (LEfSe) identifying differentially abundant bacterial taxa between groups at week 12. Horizontal bars represent LDA scores (log₁₀); taxa enriched in the MD group are shown on the right (blue), and those enriched in the control group on the left (gray). LDA score threshold = 2.0.

Beta diversity analysis demonstrated distinct clustering patterns between groups at week 12, as illustrated in Figures 2A,B. Principal coordinates analysis (PCoA) based on Bray–Curtis dissimilarity revealed substantial overlap between groups at baseline, indicating comparable community structures prior to the intervention. By week 12, the Mediterranean diet group exhibited a clear shift along the first principal coordinate axis (explaining 24.3% of variance), while the control group remained clustered near baseline positions. PERMANOVA confirmed that the dietary intervention significantly influenced overall microbiota composition (*R*^2^ = 0.089, *p* = 0.001), with the group-by-time interaction explaining 5.4% of the total variance (*p* = 0.003).

Differential abundance analysis using LEfSe identified 18 taxa that differed significantly between groups at week 12 (LDA score > 2.0), as shown in Figure 2C. The Mediterranean diet group was characterized by enrichment of several SCFA-producing genera, including *Faecalibacterium* (LDA = 4.21, *p* < 0.001), *Roseburia* (LDA = 3.87, *p* = 0.002), *Bifidobacterium* (LDA = 3.54, *p* = 0.004), and *Akkermansia* (LDA = 3.12, *p* = 0.008). At the species level, *Faecalibacterium prausnitzii*, a major butyrate producer in the human gut, showed a 2.4-fold increase in relative abundance in the Mediterranean diet group compared to baseline (*p* < 0.001), with no significant change observed in controls. The control group exhibited relatively higher abundance of *Bacteroides fragilis* (LDA = 2.89, *p* = 0.018) and *Alistipes putredinis* (LDA = 2.45, *p* = 0.032) at week 12. Temporal analysis indicated that microbial shifts were already detectable at week 6, with further divergence occurring between weeks 6 and 12, suggesting progressive remodeling of the gut ecosystem in response to sustained dietary modification.

### 3.3 Short-chain fatty acid profiles

Fecal and plasma SCFA concentrations were quantified at baseline, week 6, and week 12 to assess the metabolic consequences of dietary-induced microbiota changes. Comprehensive SCFA data are presented in Table 2.

Fecal SCFA analysis revealed significant increases in the Mediterranean diet group over the intervention period. Total fecal SCFA concentration increased from 68.4 ± 12.8 μmol/g at baseline to 82.6 ± 15.2 μmol/g at week 12, representing a 20.8% increase (*p* = 0.004). This elevation was primarily driven by increases in propionate (baseline: 14.8 ± 3.4 μmol/g; week 12: 19.6 ± 4.2 μmol/g; *p* = 0.002) and butyrate (baseline: 11.5 ± 2.8 μmol/g; week 12: 14.8 ± 3.4 μmol/g; *p* = 0.008). Fecal acetate showed a trend toward increase that did not reach statistical significance (baseline: 42.1 ± 8.2 μmol/g; week 12: 48.2 ± 9.8 μmol/g; *p* = 0.062). The control group exhibited no significant changes in fecal SCFA concentrations throughout the study period (total SCFA baseline: 67.8 ± 13.2 μmol/g; week 12: 69.1 ± 13.5 μmol/g; *p* = 0.71). The group-by-time interaction was statistically significant for total fecal SCFA (*p* = 0.008), propionate (*p* = 0.003), and butyrate (*p* = 0.012), but not for acetate (*p* = 0.09). Branched-chain SCFAs including isobutyrate, valerate, and isovalerate showed no significant changes in either group, consistent with their origin from protein rather than fiber fermentation.

**Figure 3:**
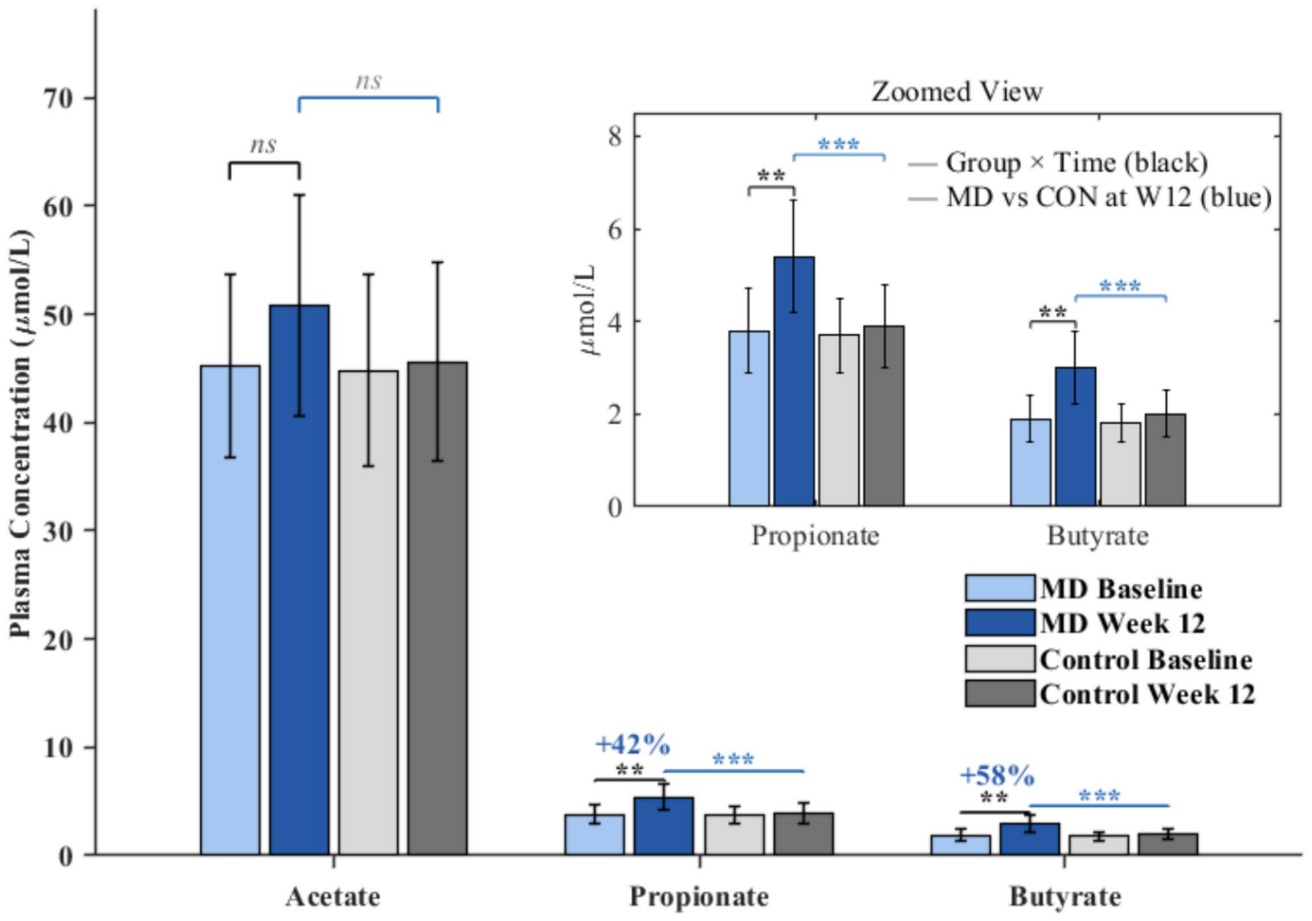
Plasma short-chain fatty acid concentrations following Mediterranean diet intervention. Plasma short-chain fatty acid (SCFA) concentrations at baseline and week 12 in Mediterranean diet (MD) and control groups. Data are presented as mean ± SD. Main panel shows acetate, propionate, and butyrate concentrations. Inset displays enlarged view of propionate and butyrate for clarity. Percentage values indicate the change from baseline to week 12 in the MD group. **p < 0.01 for group × time interaction; ns, not significant. Note that acetate showed a non-significant trend (*p* = 0.12). ***indicates *p* < 0.001.

Plasma SCFA concentrations partially mirrored the patterns observed in fecal samples, as illustrated in Figure 3. In the Mediterranean diet group, plasma propionate increased by 42.1% from 3.8 ± 0.9 μmol/L at baseline to 5.4 ± 1.2 μmol/L at week 12 (*p* = 0.005), while plasma butyrate demonstrated a 57.9% increase from 1.9 ± 0.5 μmol/L to 3.0 ± 0.8 μmol/L (*p* = 0.002). Plasma acetate showed a modest but non-significant increase of 12.4% (baseline: 45.2 ± 8.5 μmol/L; week 12: 50.8 ± 10.2 μmol/L; *p* = 0.12). The control group maintained relatively stable plasma SCFA levels, with no significant changes detected for any individual SCFA species. Considerable inter-individual variability was observed in SCFA responses, with approximately 20% of participants in the Mediterranean diet group showing minimal changes in plasma SCFA despite adequate dietary compliance, suggesting that additional factors such as baseline microbiota composition may influence metabolic responsiveness to dietary intervention.

Correlation analysis revealed significant positive associations between the abundance of SCFA-producing bacteria and circulating SCFA concentrations, although the strength of these relationships was moderate. Changes in *Faecalibacterium* relative abundance correlated positively with changes in plasma butyrate (*r* = 0.46, *p* = 0.002), consistent with the established role of *Faecalibacterium prausnitzii* as a primary butyrate producer. Changes in *Roseburia* abundance were associated with plasma butyrate (*r* = 0.38, *p* = 0.012) but showed only a trend-level association with propionate (*r* = 0.28, *p* = 0.068). The correlation between *Akkermansia* and plasma propionate did not reach significance (*r* = 0.22, *p* = 0.14). These findings suggest a mechanistic link between observed microbiota shifts and elevated circulating SCFA levels, while also highlighting the complexity of host-microbiome metabolic interactions. The effect sizes for between-group differences in SCFA changes were large for both plasma propionate (Cohen’s d = 2.09) and butyrate (d = 2.20), indicating substantial metabolic responses to the dietary intervention.

### 3.4 Exercise performance and mediation analysis

Both groups demonstrated improvements in aerobic capacity following the 12-week training program; however, the magnitude of adaptation differed between dietary conditions. Participants in the Mediterranean diet group exhibited a mean VO_2_max increase of 2.4 ± 1.6 mL/kg/min (4.4% improvement from baseline), compared to 1.3 ± 1.4 mL/kg/min (2.4% improvement) in the control group. ANCOVA adjusting for baseline VO_2_max, age, sex, and body mass index confirmed a statistically significant between-group difference (adjusted mean difference: 1.1 mL/kg/min; 95% CI: 0.3–1.9; *p* = 0.006; Cohen’s d = 0.73). Time to exhaustion increased by 9.8% in the Mediterranean diet group versus 5.2% in controls (*p* = 0.024; d = 0.58). Lactate threshold showed a trend toward greater improvement in the Mediterranean diet group (2.1% vs. 1.2%), although this difference did not reach statistical significance (*p* = 0.08; d = 0.35). Notably, considerable inter-individual variability was observed in performance responses; approximately 25% of participants in the Mediterranean diet group exhibited VO_2_max improvements of less than 1.5 mL/kg/min despite good dietary compliance, suggesting that factors beyond diet influence the magnitude of training adaptation.

**Figure 4:**
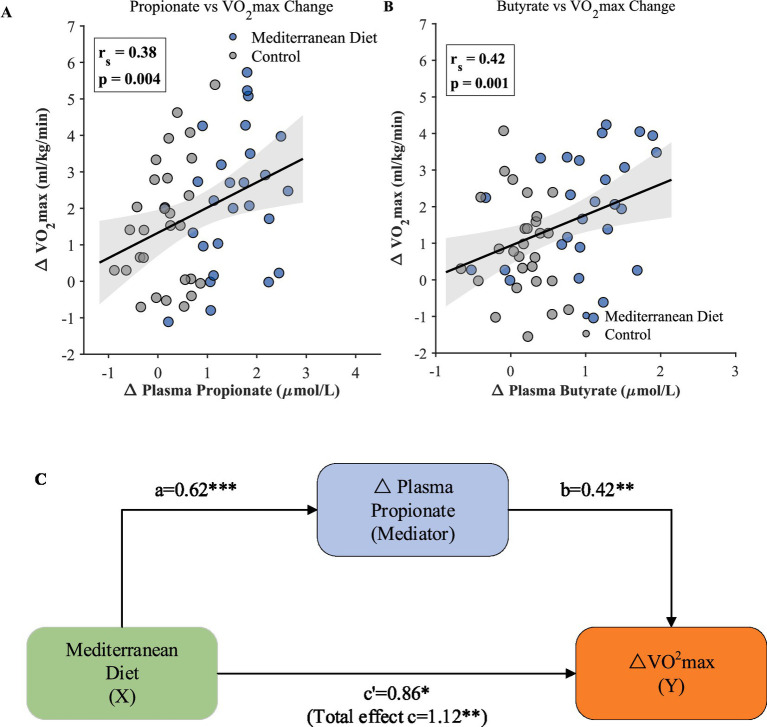
Associations between short-chain fatty acids and endurance performance. **(A,B)** Spearman correlations between changes in plasma propionate (left panel; *r*_s_ = 0.38, *p* = 0.004) and butyrate (right panel; *r*_s_ = 0.42, *p* = 0.001) concentrations and changes in maximal oxygen uptake (VO_2_max). Solid lines represent linear regression; shaded areas indicate 95% confidence intervals. Blue circles: Mediterranean diet group; gray circles: control group. **(C)** Mediation analysis examining the role of plasma propionate in mediating the effect of Mediterranean diet on VO_2_max improvement. Path coefficients are unstandardized regression coefficients (*a* = 0.62, *b* = 0.42, *c* = 1.12, *c*’ = 0.86). The indirect effect (*a* × *b* = 0.26; 95% CI: 0.05–0.58) indicates that plasma propionate mediated approximately 23% of the total dietary effect on VO_2_max. Bootstrap resampling: 5000 iterations. *p < 0.05, **p < 0.01, ***p < 0.001.

Correlation analyses revealed moderate positive associations between changes in circulating SCFA concentrations and improvements in endurance performance, as depicted in Figures 4A,B. Changes in plasma propionate were positively correlated with VO_2_max improvement (Spearman *r* = 0.38, *p* = 0.004), as were changes in plasma butyrate (*r* = 0.42, *p* = 0.001). These correlations remained significant but attenuated after adjusting for dietary group assignment (partial *r* = 0.29 and 0.33, respectively; both *p* < 0.05), indicating that part of the association was explained by group differences. Plasma acetate changes showed a weak and non-significant association with VO_2_max improvement (*r* = 0.21, *p* = 0.13). Correlations between SCFA changes and secondary performance outcomes are summarized in Table 4; associations with time to exhaustion were generally weaker than those with VO_2_max, and correlations with lactate threshold largely failed to reach significance.

**Table 4:** Correlations between SCFA changes and performance outcomes.

Variable	ΔVO_2_max	ΔTime to exhaustion	ΔLactate threshold
ΔPlasma propionate	0.38**	0.31*	0.24
ΔPlasma butyrate	0.42**	0.35**	0.26
ΔPlasma acetate	0.21	0.18	0.12
ΔFecal total SCFA	0.34*	0.28*	0.19

Values are Spearman correlation coefficients (*r*). *p < 0.05; **p < 0.01. Δ indicates change from baseline to week 12. Non-significant correlations (*p* ≥ 0.05) are shown without asterisks.

Mediation analysis was conducted to quantify the extent to which plasma propionate mediated the effect of Mediterranean diet intervention on VO_2_max improvement, with results illustrated in Figure 4C. The analysis confirmed significant paths from dietary intervention to plasma propionate change (path a = 0.62, *p* < 0.001) and from propionate change to VO_2_max improvement controlling for diet (path b = 0.42, *p* = 0.008). The total effect of Mediterranean diet on VO_2_max (path c = 1.12, *p* = 0.006) was partially attenuated when plasma propionate was included as a mediator (direct effect c’ = 0.86, *p* = 0.028). Bootstrap analysis with 5,000 iterations yielded a significant indirect effect of 0.26 (95% CI: 0.05–0.58), indicating that plasma propionate changes accounted for approximately 23% of the total dietary effect on VO_2_max improvement. A supplementary mediation model incorporating plasma butyrate as the mediator produced similar but slightly weaker results, with butyrate explaining 18% of the intervention effect (indirect effect = 0.20; 95% CI: 0.02–0.52). These findings suggest that SCFA-mediated mechanisms contribute to, but do not fully explain, the enhanced training adaptation observed with Mediterranean diet intervention.

## 4 Discussion

It was shown within this study that a Mediterranean diet intervention affects the intestinal microbiota composition and raises serum levels of SCFAs in endurance athletes, and such a metabolic change partially contributes to training adaptation. The observed increase in SCFA-producing bacteria, along with plasma concentrations of propionate and butyrate and their associations with improvements in aerobic capacity, provide evidence to support a mechanism for gut-muscle axis effects on endurance exercise performance (5, 21, 22). These findings have widened the existing body of knowledge about the associations among diet, microbiota, and performance, and may therefore lay the groundwork for designing nutritional strategies aiming to modulate microbial metabolism for optimizing performances (8, 14).

The microbiota changes are consistent with those reported in other Mediterranean diet intervention studies. The enrichment of *Faecalibacterium*, *Roseburia*, and *Bifidobacterium* is consistent with the results from systematic reviews, according to which a high dietary fiber intake encourages the proliferation of saccharolytic bacteria (10, 23). What is remarkable about the current data is the fact that such changes in microbiota have been recorded even in highly trained athletes, considering that their microbial diversity is higher due to the distinct microbial profiles found in athletes as opposed to sedentary individuals (4, 24, 25). The simultaneous enrichment of *Akkermansia* is in accordance with findings of Meslier et al. (11) regarding the Mediterranean diet-induced increase of this beneficial mucin-degrading bacteria; whereas decreases in *Bilophila* and protein-fermenting bacteria represent differential substrate availability based on dietary pattern (26).

Increase in plasma concentrations of SCFA is a physiological indicator of a link between the effects of microbiota change and systemic physiology on a metabolic level. While the determination of fecal SCFA content indicates colonic fermentation, plasma levels more specifically indicate the concentration available to peripheral tissues, including skeletal muscle (27). The role of SCFAs in modulating skeletal muscle metabolism is summarized in detail by Frampton et al. (5), who pointed to activation of the G-protein coupled receptor, histone deacetylase inhibition, and availability of metabolic substrates as being the key pathways. Perhaps, the preferential increase in butyrate and propionate over acetate is due to the stronger signaling activity through GPR41 and GPR43 receptors, whereas acetate is mainly utilized as an oxidation substrate (7). The observed interpersonal differences, whereby 20% of participants showed small responses to SCFAs despite their confirmed adherence to the diet, suggest that individual differences in the baseline composition of their gut microbiota influence their responsiveness to the diet (28). Moreover, these findings are in line with studies exploring personal responses to standardized diets in precision nutrition studies (29). These findings can be more explicitly framed within the emerging concept of “metabolic responders” versus “non-responders” to dietary microbiota-targeted interventions. In the present cohort, the approximately 20% of participants who exhibited minimal plasma SCFA elevation despite confirmed dietary adherence may represent individuals whose baseline microbiota lacked sufficient SCFA-producing capacity to respond to increased fiber substrate. This interpretation is consistent with precision nutrition paradigms suggesting that baseline gut microbiota composition may serve as a predictive biomarker of individual responsiveness to fiber-rich dietary interventions. Future studies incorporating pre-intervention microbiota profiling could use such information to identify likely responders in advance, thereby enabling more targeted and individualized application of Mediterranean diet strategies in endurance athlete populations.

The positive correlations between the change of SCFA and the improvement of VO_2_max support the hypothesis of the gut-muscle axis proposed by Scheiman et al. (22). The groundbreaking study conducted by the scientists illustrated that *Veillonella atypica*, which was isolated from marathon runners after exercise, produces lactate to propionate conversion and stimulates treadmill exercise performance in mice (22). Subsequent metagenomic analysis by Fontana et al. confirmed this result, finding the gut microbiomes of elite athletes to be enriched for genes coding for the production of SCFAs. Related research by Kulecka et al. (4) has shown the presence of specific compositions of commensal bacteria in the best endurance athletes (6). The effect of diet on aerobic adaptation was shown to be mediated, with the plasma concentration of propionate mediating about 23% of the effect. Several non-SCFA mechanisms could account for observed performance benefits, which include anti-inflammatory actions mediated by the improvement of gut barriers function (30), stimulation of mitochondrial biogenesis driven by polyphenols (31), and dietary nitrate supplementation benefits related to increased nitric oxide bioavailability and exercise efficiency (32). The observed partial mediation appears to be biologically valid and consistent with the complexity of relationships between dietary factors and performance described by recent integrative reviews (8, 33).

The difference between groups for the change in VO_2_max of around 1.1 mL/kg/min. Represents a 2.0% relative improvement, which approaches the lower boundary of the reported test–retest measurement error for VO2max (typically 3–5%). Consequently, the practical competitive significance of this difference should be interpreted cautiously, as the observed effect may partially reflect measurement imprecision rather than a true physiological adaptation. Additionally, the single-blind design and potential learning effects across repeated maximal tests may have contributed to the between-group difference, and these sources of bias cannot be entirely excluded. Bassett and Howley (1) have picked out VO_2_max as being of prime importance for endurance exercise performance, while meta-analyses conducted by Milanović et al. (16) found an increase of 5 to 20% according to initial level of fitness and method of exercise. A systematic review carried out by Crowley et al. (34) highlights the importance of training intensity to have a positive impact on aerobic exercise adaptation. It is important to note that the finding indicating that the modification of dietary patterns increases VO_2_max independently of training further supports the additive effect of combining the two, which is consistent with periodization principles. The large level of inter-individual differences in performance responses, in keeping with the findings of Burke et al. (3) regarding VO_2_max trainability, underlines the need for individual assessments in the practical field of sports nutrition.

There could be a number of mechanisms for the association between SCFA and performance in this study. Bongiovanni et al. (27) postulated a possible ergogenic use for SCFAs in improving substrate availability, efficiency of metabolism, and anti-inflammatory properties. Ong et al. highlight the beneficial effects of SCFA supplementation on athletic health and exercise performance, whereas Chen et al. (8) focus on bidirectional signaling in the gut-muscle axis. Propionate and butyrate activate AMP-activated protein kinase, a mechanism potentially enhancing mitochondrial biogenesis and oxidation in skeletal muscle (7). Gao et al. (7) found the ability of butyrate to increase the sensitivity of the body to insulin and boost energy expenditure by improving oxidative metabolism, while Pan et al. identified the potency of acetic acid to improve endurance performance by increasing the oxidative qualities of skeletal muscle properties. Recently, Liu et al. (6) showed that the SCFAs induce positive muscle hypertrophy and function using activation of the mTOR pathway. This mechanistic evidence is helping drive research on SCFAs as potential mediators of positive performance responses induced by diet manipulation (8).

Certain methodological issues need to be kept in mind while analyzing the results. The single-blind design poses the risk of performance bias with regard to both adherence and subjective outcomes, as was pointed out in previous dietary intervention studies (12). Regarding duration, the 12-week study period used is adequate to study the changes in the microbiota, but it may not be able to assess the sustained effect following the withdrawal of the dietary intervention. The limited study sample of young endurance athletes prevents the generalization of the results, and recently published studies by Shalmon et al. have identified discipline-related differences in the microbiome composition of athletes (25). The analysis pipeline employed standard approaches including QIIME2 (18), DADA2 (19), and LEfSe (20), consistent with recent methodological recommendations (35). Future studies should apply metagenomic sequencing for the determination of functional capacity and extend the timeframe of interventions for the assessment of persistence of studied effects. Several additional limitations warrant acknowledgment. First, the pragmatic nature of the control condition—where participants maintained their habitual diet while receiving general nutritional guidance—may have reduced dietary contrast between groups if fiber intake or Mediterranean diet adherence in the control group drifted upward over the intervention period. Based on three-day food records and MEDAS scores, mean dietary fiber intake in the control group was 17.8 ± 5.2 g/day at T0 and 18.5 ± 4.9 g/day at T12 (*p* = 0.31), and MEDAS scores were 5.6 ± 2.1 at T0 and 5.9 ± 2.0 at T12 (*p* = 0.42), suggesting that the dietary separation between groups was maintained throughout the study. To the extent that any convergence occurred, the between-group effect estimates reported here should be regarded as conservative. Second, the absence of direct skeletal muscle measurements—such as markers of mitochondrial biogenesis (e.g., PGC-1α, citrate synthase activity) or AMPK signaling—means that the proposed mechanism linking SCFA elevation to VO₂max adaptation cannot be directly confirmed; the microbiota-mediated pathway remains biologically plausible but mechanistically inferred. Third, the Mediterranean diet is a complex dietary pattern encompassing multiple bioactive components including dietary fiber, polyphenols, and unsaturated fatty acids, each of which may independently influence gut microbiota and metabolic outcomes; the current design does not permit isolation of the specific contribution of any single dietary component.

The results have significant implications within sports nutrition practice. Griffiths et al. highlight the Mediterranean diet as a tool for maximizing health outcomes in competitive athletes (17), whilst Mancin et al. (15) illustrate how variants of the Mediterranean diet have been shown to affect the microbiome of the athlete. The enhancement of Mediterranean-style dietary patterns could have performance gains in addition to the traditional optimal nutrient macronutrient strategies as identified in consensus statements, though the variability evident suggest the importance of individual assessments of microbiota and metabolism (28). In the present study, meaningful between-group differences in plasma SCFA concentrations were already detectable at week 6 (T6), suggesting that at least 6 weeks of dietary adherence may be required before appreciable SCFA elevations occur; however, as longitudinal microbiota profiling beyond T0 and T12 was not conducted, this inference is preliminary and requires confirmation in studies with denser sampling intervals. If replicated, this finding would imply that Mediterranean diet interventions targeting SCFA-mediated adaptation should be initiated well in advance of key competition periods. A possible integration of microbial analyses with athlete monitoring programs could make personalized nutritional recommendations on the basis of individual microbial compositions.

The findings have various implications for sports nutrition practice. The Mediterranean dietary habits could provide certain performance benefits, other than traditional macronutrient ratios, due to their favorable impact on microbial metabolism. However, the observed large variations between individuals, with regard to both microbial composition and performance, emphasize the needs for individualized nutritional practice. Future research should incorporate the use of metagenomic and metabolomic analyses to better understand the underlying mechanisms among diet-microbiota-performance associations. It would also be important to extend the duration of the interventions so that the longevity of the response can be tested. Microbiome data could be used to tailor the diet of athletes.

## 5 Conclusion

This randomized controlled trial provides evidence that after 12 weeks of Mediterranean dietary intervention, the composition of gut microbes is significantly changed in endurance athletes by enriching their population of SCFA-producing bacteria such as *Faecalibacterium*, *Roseburia*, and *Bifidobacterium*. These changes in the microbial communities were accompanied by the increased plasma levels of propionate and butyrate, which showed moderate positive correlations to the improvements observed in maximal oxygen uptake. The mediation analysis showed that plasma propionate was associated with about 23% mediation of the VO_2_max. Response, suggesting that SCFA-mediated pathways may contribute to, but do not fully account for, the observed training-related improvements. Collectively, these results support the gut-muscle axis as a modifiable target through Mediterranean dietary intervention, providing a mechanistic basis for integrating microbiota-targeted nutritional strategies into endurance training practice.

## Data Availability

The data presented in the study are deposited in the Zenodo repository, accession number 18846564 (DOI: 10.5281/zenodo.18846564).
